# Robust, Integrated Computational Control of NMR Experiments to Achieve Optimal Assignment by ADAPT-NMR

**DOI:** 10.1371/journal.pone.0033173

**Published:** 2012-03-12

**Authors:** Arash Bahrami, Marco Tonelli, Sarata C. Sahu, Kiran K. Singarapu, Hamid R. Eghbalnia, John L. Markley

**Affiliations:** 1 National Magnetic Resonance Facility at Madison, Biochemistry Department, University of Wisconsin - Madison, Madison, Wisconsin, United States of America; 2 Center for Eukaryotic Structural Genomics, University of Wisconsin-Madison, Madison, Wisconsin, United States of America; 3 Department of Molecular and Cellular Physiology, University of Cincinnati, Cincinnati, Ohio, United States of America; National Institute for Medical Research, Medical Research Council, London, United Kingdom

## Abstract

ADAPT-NMR (Assignment-directed Data collection Algorithm utilizing a Probabilistic Toolkit in NMR) represents a groundbreaking prototype for automated protein structure determination by nuclear magnetic resonance (NMR) spectroscopy. With a [^13^C,^15^N]-labeled protein sample loaded into the NMR spectrometer, ADAPT-NMR delivers complete backbone resonance assignments and secondary structure in an optimal fashion without human intervention. ADAPT-NMR achieves this by implementing a strategy in which the goal of optimal assignment in each step determines the subsequent step by analyzing the current sum of available data. ADAPT-NMR is the first iterative and fully automated approach designed specifically for the optimal assignment of proteins with fast data collection as a byproduct of this goal. ADAPT-NMR evaluates the current spectral information, and uses a goal-directed objective function to select the optimal next data collection step(s) and then directs the NMR spectrometer to collect the selected data set. ADAPT-NMR extracts peak positions from the newly collected data and uses this information in updating the analysis resonance assignments and secondary structure. The goal-directed objective function then defines the next data collection step. The procedure continues until the collected data support comprehensive peak identification, resonance assignments at the desired level of completeness, and protein secondary structure. We present test cases in which ADAPT-NMR achieved results in two days or less that would have taken two months or more by manual approaches.

## Introduction

A goal-directed experimental strategy can be defined as one that optimizes each new experimental step by analyzing the current sum of available results with the aim of achieving a particular goal. In principle, such a strategy should be superior to ones in which data are collected first, either by conventional or fast methods, and analyzed later. The idea of focusing on an end goal to guide data collection could be broadly applicable to many domains of investigation. We describe here a proof of concept implementation of this strategy to the collection and analysis of protein NMR data with the goal of achieving complete resonance assignments of the type required for automated structure determination. Our approach, ADAPT-NMR (Assignment-directed Data collection Algorithm utilizing a Probabilistic Toolkit in NMR), successfully navigates a large set of experimental options on the basis of iterative analysis of the current data and achieves efficient and complete assignments and secondary structure determination.

**Figure 1:**
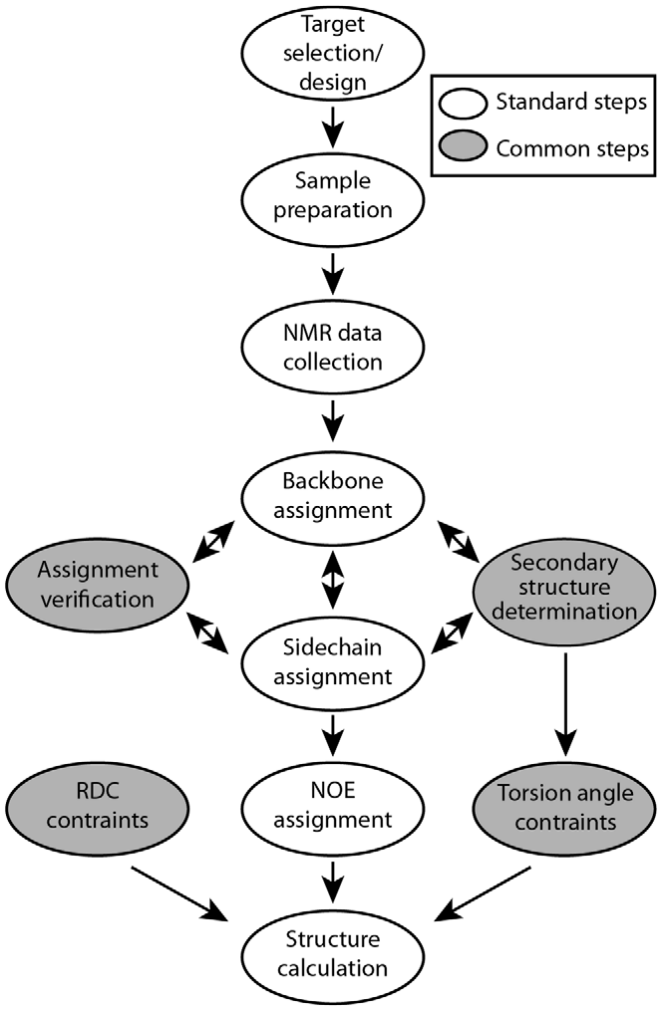
Conventional steps in protein structure determination by solution state NMR spectroscopy.

The initial stage in solution-state NMR spectroscopy of proteins concerns the production of labeled molecules and the identification of suitable solution conditions for data collection. These steps are analogous to the production of protein and suitably diffracting crystals for X-ray crystallography. Whereas, with crystallography, the subsequent data collection and analysis steps leading to structure determination are fairly standardized and automated, this is not yet the case with protein NMR spectroscopy. Typically, several multinuclear, multidimensional NMR data sets are collected and subsequently analyzed in separate steps leading to a structure (Figure 1). Each of these steps has been automated to some extent [1], [2], [3], [4], [5], [6], [7]; and in some cases, multiple steps have been pipelined to work sequentially [8], [9]. However, a software pipeline is not adept at emulating the iterative nature of structure determinations performed by human experts. As a result, manual intervention in data analysis continues to be one of the main bottlenecks in structure determination by NMR. ADAPT-NMR is the first iterative and fully automated approach designed specifically for the optimal assignment of proteins. The increased efficiency results from more rapid data collection, data analysis, and data verification.

## Methods

### Approach

**Figure 2:**
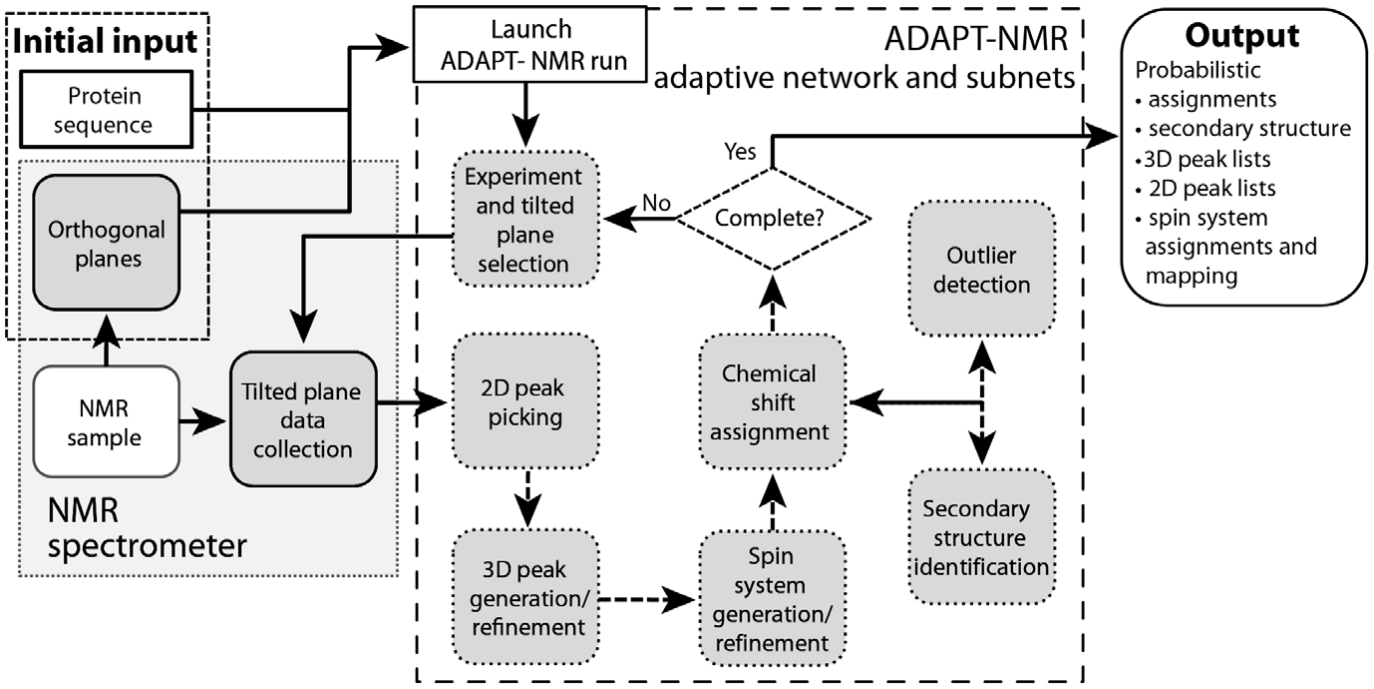
Overview of ADAPT NMR. The shaded rectangle at the left shows the sample and operations carried out by the NMR spectrometer. Initial input is indicated in the upper left corner. The dashed line encloses components of the probabilistic network. The ADAPT-NMR output is listed at the right.

ADAPT-NMR builds a fully probabilistic, yet computationally tractable, network capable of dynamically representing interrelationships among assigned attributes. Our development of this goal-directed approach required a fundamental reevaluation of our prior paradigm for NMR data collection and analysis [10], [11]. ADAPT-NMR uses a probabilistic goal-seeking approach in which a set of cooperating probabilistic sub-networks, each implementing their own computational model, drive toward the optimal fashion by controlling the flow of experiments (Figure 2).

The goal-seeking tasks select two parameters at each iteration: a) the 3D NMR (^1^H, ^13^C, and ^15^N) experiment to be conducted in the next *step*, and b) the subspace (tilt angle that combines ^13^C and ^15^N frequencies to reduce data collection from 3D to 2D) used in data collection. The subspace can be considered as the projection of 3D spectra into 2D tilted planes. The cooperating sub-networks enforce three key conditions designed to enhance the stability and reliability of the global network: a) probabilistic representation, b) concise probabilistic communication, and c) simplified domain decomposition. The choice of these conditions was motivated by practical experience with Bayesian updating approaches. Condition a) requires that each subnet must represent its state by an ensemble of probabilistic variables. Condition b) requires that subnets communicate with one another by means of probability distributions over all the variables they share, and Condition c) maintains network robustness (See Text S1).

The architecture of the network integrates an extensive pseudo-energetic model (analogous to those used in biophysics and statistical mechanics) for each subnet by using a number of mathematical and machine learning [10], [12] techniques. With each step in data collection and analysis, the network evolves toward an ensemble of states – a *configuration* – that represents the assignment of NMR resonances (chemical shifts) and secondary structural elements to groups in the covalent structure of the protein.

The pseudo-energetic system is represented by a canonical ensemble, in which the probability of each configuration *p*_i_ (corresponding to microscopic state ‘*s*’) is given by the Boltzmann distribution
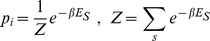
(1)where *β* resembles the thermodynamic variable (determined empirically), and *Z* is the canonical partition function. *E_S_*, the energy of microstate *s*, is the sum of individual and interaction potentials. In our model, individual potentials represent prior information, such as NMR chemical shift distributions and prior probabilities for the most recent model for peak lists, resonance assignments, and secondary structure probabilities. The interaction potentials, on the other hand, represent constraints and the consistency of variable choices. The total energy of the network in ADAPT-NMR is written as a sum of single, pairwise, and triple-wise interactions among network microstates:
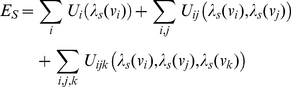
(2)where *λ_s_(v_i_)* represents the state of the probabilistic variable *v_i_*, *U_i_* represent individual potentials, and *U_ij_* and U*_ijk_*, respectively, represent pair-wise and triple-wise interaction potentials. The use of triple-wise interactions is unique to our definition of the statistical model. In ADAPT-NMR, rather than seeking a single solution, which would necessitate the identification of the unique configuration that minimizes the total energy, we determine marginal probabilities for every probabilistic variable.

Approximation of the ground state, a state where probabilities are effectively stationary, relies on the implementation of algorithms of the kind used in graphical models [13], [14], [15]. We use the factor graph representation [16] because it is computationally efficient and it has been applied successfully in diverse areas of information technology [17]. In the factor graph representation [18], our pseudo-energetic model transforms to factorized local functions in which the probability for a configuration *s* can be described as:
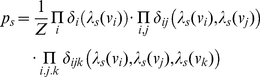
(3)where the factors *δ_i_*, *δ_ij_*, and *δ_ijk_* can be calculated from pseudo-energy terms as: 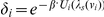, 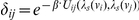, and 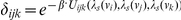


However, the resulting computational structure does not guarantee convergence to the ground state [13], [14], [15]. To overcome this, we have developed specialized algorithms in ADAPT-NMR. Detailed description of the ideas and algorithms can be found in the Text S1.

### Algorithm

The algorithm is briefly summarized here (see the Text S1 for complete details). The ADAPT-NMR iteration starts with the amino acid sequence of the protein and 2D NMR data sets ^1^H-^15^N (^15^N-HSQC) and ^1^H-^13^C. These data sets serve as “orthogonal projections” of conventional 3D NMR spectra. Then ADAPT-NMR applies an advanced automated peak identification algorithm, and generates probabilistic “spin systems”. A spin system is defined as a group of peaks that are most likely belong to the same amino acid in the protein sequence. At this level, on-the-fly evaluations of the spin systems determine which experiment and projection (tilted plane) should be collected next. The iteration continues until the spin system quality is good enough for initial calculations of sequence-specific resonance assignments and secondary structure. Thereafter, an extended network, which takes into consideration spin systems, chemical shift assignments, and secondary structure, selects the next experiment and tilted plane. The iteration continues until the desired completeness of chemical shift assignments is achieved (Figure 2).

#### Spectral Acquisition

The optimum spectra (as determined in the optimization step) are collected by ADAPT-NMR, and are classified as *S_i,j_*, where ‘*i*’ is the experiment identifier (for example HNCA) and ‘*j*’ is the tilt angle (projection angle). Tilted angle spectra are generally collected in pairs (*S_i,j_* and *S_i,-j_*).

#### Spectral Processing

The key derived measure in this step is the “conclusive probability” for each identified spectral peak, which is defined as the probability that a peak represents a real peak as opposed to an artifact or noise peak. ADAPT-NMR imports the most recently collected spectral data, co-registers all peaks by aligning all spectra, and peak picks spectra by an algorithm that assigns a probability to each peak on the basis of the noise level, peak intensity, the number of the residues in the protein, and the experiment type. Every 2D peak maintains a set of specific attributes (or properties), e.g., frequency coordinates, intensity, volume, possible back-projected 3D peak candidate, and priority weight.

ADAPT-NMR generates a candidate 3D peak with numerous attributes from every pair of peaks present in the orthogonal planes that have a common ^1^H chemical shift (within a tolerance). The 3D peak lists are updated after each step of data collection.

#### Spin System Generation and Update

ADAPT-NMR applies the pseudo-energetic model presented above and an iterative update algorithm to derive probabilistic spin systems from available peak lists. Spin system objects are initialized from ^15^N-HSQC peaks and have multiple attributes and properties, including eight fields that represent the chemical shifts of different classes of nuclei: *^13^C^α^_(i-1)_*, *^13^C^β^_(i-1)_*, *^13^C′_(i-1)_*, *^1^H_(i)_*, *^15^N_(i)_*, *^13^C^α^_(i)_*, *^13^C^β^_(i)_*, and *^13^C′_(i)_*, where *(i-1)* denotes the chemical shift of the previous residue. Each field is a probabilistic variable that might have multiple chemical shift choices. The chemical shift choices and their probabilities are calculated in the probabilistic network on the basis of 3D peak attributes. A “null” state for matching is provided in order to represent the probability that no chemical shift in the data could be matched with the field. Null is a possible state for almost every probabilistic variable in ADAPT-NMR.

All attributes of spin systems are updated after each round of iteration and data collection. New spin systems are added if high probability peaks cannot be associated with any ^15^N-HSQC peaks. An important attribute of spin systems is “the probability of overlap”. In overlapped spectral regions, multiple spin systems may originate from a single ^15^N-HSQC peak. A probabilistic support vector machine (SVM) [12] has been trained to continuously evaluate this probability. If the probability of overlap surpasses a threshold, the spin system is split. This feature of ADAPT-NMR has substantially improved the assignment quality of crowded spectral regions, and it is absolutely crucial for larger proteins.

#### Update Assignment

If the quality of spin systems is lower than a pre-selected threshold, the algorithm transfers control to the optimization step (described below). Otherwise, in the assignment step, probabilities for chemical shift assignments, secondary structure states, and outlier chemical shift values are determined. The core elements of this step were initially designed as part of the PINE algorithm [10]. However, extensions resulting from key insights gained from earlier work have resulted in modifications that led to substantial improvements in the quality of the assignment. These improvements are described in the Text S1.

#### Optimization Step

In the optimization step, ADAPT-NMR selects the next experiment and the next projection (tilted plane) for maximally informative data collection by utilizing information theory [19]. The first step is to use the level of “uncertainty” in the sense of information theory in order to identify the nuclei in the spin systems that are “weakest links” in the assignment process. This step pinpoints specific spin systems and candidate nuclei (fields) that have not been assigned uniquely and, therefore, have more “uncertainty”. In the next step ADAPT-NMR determines the optimal experiment that is expected to maximize the information gain. This step optimally selects the tilted plane with minimal overlap for the pinpointed peaks. The selection set of tilted planes involves a search and prediction algorithm for all angles from 1° to 89° with increments of 1°, excluding planes that have already been collected. Data collection for the specified experiment and the specified tilted plane takes place without any user intervention.

#### Evaluation Decision

The latest results are generated (including the latest output files for chemical shift assignments, secondary structure, outliers, spin systems, 2D and 3D peak lists and their associated probabilities), and the overall assignment score is calculated. If this score falls below the specified target, the utility of further data collection is evaluated. Factors in this decision include whether the maximum numbers of tilted planes specified for each experiment have been collected, the quality factors, the number of residues, and the level of improvement in the assignment score from the previous iteration. If further data collection is not advised or if the assignment target score has been reached, a report is written, and the process terminates. Otherwise, data collection continues.

### Software

ADAPT-NMR consists of more than 60,000 lines of MATLAB code, shell scripts, and the Varian (Bruker) macro languages. The MATLAB code includes all steps described in the algorithm section except for spectral acquisition and is also available as a single executable file for multiple platforms. Programs written in the Varian (Bruker) macro languages control data acquisition by the spectrometer, and the shell script macro provides the interface between data collection and data analysis. The automated process is started by executing a macro from within the spectrometer interface (VnmrJ/Topspin), the tilted planes for the various 3D spectra are then collected, processed and analyzed on-the-fly and without any manual intervention. The output from the analysis module is automatically fed back into the spectrometer to direct data collection.

Execution of the data analysis module normally takes between a few seconds up to 2 min, depending on the complexity of the data and speed of the processor. These times are far shorter than the time required for tilted plane data acquisition (10–15 min on average). The complete analysis of 2D planes, 3D peaks, spin systems, and the complete assignment and secondary structure determination of the protein is provided by ADAPT-NMR in numerous formats. The ADAPT-NMR software package and a comprehensive description page is publically available at http://pine.nmrfam.wisc.edu/ADAPT-NMR/.

### Protein sample

We used three small proteins, human ubiquitin, chlorella ubiquitin, and brazzein RI (RI insertion between L18 and A19 for WT brazzein) to test the ADAPT-NMR algorithm as it was being developed. Subsequently, we tested the algorithm on three more proteins. The sterol carrier protein-2 from *Aedes aegypti* (AeSCP2) contained bound palmitate [20]. HSP12 is an intrinsically disordered 12 kDa heat shock protein [21], and SOX2(39–118) is the HMG box (DNA binding domain) from the 317-residue human transcription factor. Details regarding the samples and experimental conditions are provided in Table S1.

### NMR data collection

Spectra were collected on Varian (Agilent) VNMRS spectrometers equipped with a cryogenic probe. Data for intrinsically disordered protein HSP12 were collected at 900 MHz (^1^H frequency) because of high peak overlap; data for all other proteins were collected at 600 MHz. All spectra were recorded at 25°C except for brazzein RI, which was at 37°C, and SOX2, which was at 15°C. Automated data collection and analysis utilized six 3D experiments collected as 2D planes: HNCO, HN(CO)CA, HN(CA) CO, HNCA, CBCA(CO)NH, and HN(CA)CB. For each protein, a ^15^N-HSQC spectrum was collected and used as the 90° orthogonal plane and 2D ^1^H-^13^C planes were collected and used as the 0° orthogonal planes. Although not required by the ADAPT-NMR algorithm, initial collection of orthogonal plane data for visual verification of the overall quality of the spectra is the recommended procedure. The pulse programs for these experiments were taken from BioPack (Varian/Agilent) and adapted for reduced dimensionality data collection as previously described. All orthogonal and tilted planes were processed automatically by ADAPT-NMR with NMRpipe software [22].

### Flexibility and customization

ADAPT-NMR has the flexibility to process multiple experiments and planes at each round of the iteration, even if they have not been suggested in previous iterations. It also continuously saves the latest status of the project. These features enable “restarts”, for example in cases where the process has been interrupted for unexpected reasons, or where data collection by specific experiments or specific angles is impractical. The default initial settings of parameters for data collection and analysis have been optimized through testing on a number of proteins. The initial values are dynamically optimized by the algorithm during the data collection process. For example, the noise threshold level for the peak picking algorithm is modified on the basis of the threshold level used for the previous plane and the expected number of peaks. ADAPT-NMR gives users the ability to manually revise all parameters used in data collection and analysis.

## Results and Discussion

We have implemented the ADAPT-NMR algorithm on Varian and Bruker spectrometers at the National Magnetic Resonance Facility at Madison (NMRFAM). We evaluated its performance (accuracy of assignment, accuracy of the secondary structure prediction, and the total time of data collection and data analysis) on six proteins labeled uniformly with ^13^C and ^15^N that have been studied in our laboratory (Table 1). The tilted angles and experiments selected on-the-fly by ADAPT-NMR for data collection and other experimental details are provided in *Table S1*.

**Table 1:** Results from ADAPT-NMR data collection and backbone analysis of six proteins.

Protein name	Amino acid residues	Time for data collection and analysis	Completeness of chemical shift assignments	Accuracy of chemical shift assignments	Accuracy of secondary structure predictions	wwPDB and/or BMRB deposition [reference]
Brazzein (RI)	54	17 h	98%	100%	100%	2KGQ, 5296 [24]
Ubiquitin (human)	76	13 h	97%	100%	100%	17769 (a)
Ubiquitin (*Chlorella*)	76	15 h	100%	100%	100%	17730 (a)
SOX2 (39–118)	81	55 h	98%	100%	100%	2LE4, 17691 (a)
AeSCP2 (complex with palmitate)	106	39 h	98%	100%	100%	2KSI, 16665 [20]
HSP12 (intrinsically disordered)	109	17 h	99%	98%	100%	17483[21]

a This work.

For proteins ranging from 54 residues to 109 residues, the total time for data collection (for orthogonal and tilted planes), assignment, and secondary structure designation ranged from 13 hours to 55 hours (Table 1) – a significant reduction in time and effort. No manual intervention was required, and the quality of assignment exceeded that normally required for structure calculation. To evaluate accuracy, we compared the ADAPT-NMR results with chemical shift assignments achieved by separate approaches and the secondary structure from coordinates deposited in PDB, when available. The assignments deposited in BMRB associated with a PDB structure have benefited from the later steps of structure calculation (e.g. NOE assignments). In the case of four proteins (ubiquitin (human), RI-brazzein, HSP12, and AeSCP2), we carefully peak picked and assigned the traditional 3D spectra manually; in all cases the level of completeness achieved by ADAPT-NMR equaled that of the manual backbone assignment. ADAPT-NMR yielded higher accuracy of assignment in less than one-half the time (Table S2), when compared with our previous pipelined approach to automation. The SOX2(39–118) structure (Figure S1) (PDB accession code 2LE4) was determined solely on the basis of the ADAPT-NMR assignments (BMRB accession code 17691).

The fact that ADAPT-NMR has access to the actual spectra and can dynamically adjust peak picking so as to optimize the assignment of spin systems gives it an advantage over automated assignment tools that deal with peak lists or spin systems. ADAPT-NMR represents a major step toward a fully automated approach for protein structure determination by NMR. Although the ADAPT-NMR algorithm has been described here as sequential, it is important to note that the implementation of the algorithm executes the data collection and data analysis steps in parallel so that subsequent steps, including assignment, do not have to wait for the data collection to be completed.

The study of aggregated, disordered, and unstable proteins has been consistently a challenge in NMR spectroscopy. Fast data collection by ADAPT-NMR might be particularly helpful in certain unstable samples (for example, samples that are stable for one or two days.). Furthermore, “auto-adjustments” have been designed in the ADAPT-NMR algorithm to manage mild aggregation or proteins with small disordered regions. Examples of these adjustments include spin system splitting and iterative peak analysis as described in the algorithm section. However, in the case of severely aggregated proteins or spectra with large exchange broadening, reduced dimensionality methods like ADAPT-NMR are not generally desirable. The recommended manual screening of orthogonal planes prior to launching ADAPT-NMR serves to detect these instances. In addition, various quality measurements are executed during the data collection, and the process will stop if they do not satisfy some minimum thresholds.

A visualization tool being developed for ADAPT-NMR has a user interface that permits manual data analysis (for example, editing of the peaks picked) either on-the-fly or as a post-processing step. After each manual change, ADAPT-NMR updates the probabilistic network, and adjusts the outputs accordingly. We expect that this visualization tool will be particularly helpful with larger or disordered proteins that prove not to be amenable to the fully-automated data collection and analysis approach.

ADAPT-NMR is readily extensible, and we plan to develop versions that include other steps of structure determination. ADAPT-NMR currently accepts side chain peak lists as an optional input and provides full side chain assignment (Table S3). However the goal is to include on-the-fly data collection and analysis of side chain and NOE data. It will be relatively easy to collect less crowded side chain experiments such as HBHA(CO)NH and C(CO)NH by the reduced dimensionality method; however, more complicated spectra (e.g., HCCH-TOCSY) normally are not amenable to reduced dimensionality collection. The on-the-fly algorithm can be programmed to decide whether data should be collected by full 3D or reduced dimensionality. The addition of these data types, particularly 3D ^15^N- and ^13^C-NOESY, should enable ADAPT-NMR to handle larger proteins. Such extensions are achievable, because each sub-network performs the inference task separately. ADAPT-NMR can be readily integrated in an iterative fashion with structure calculation programs such as CYANA [1] or CS-Rosetta [23].

## Supporting Information

Text S1 **It discusses the robustness of the ADAPT-NMR approach and contains a complete description of the algorithm.**
(DOC)

Figure S1 **NMR solution structure of human SOX2(39–118).** This structure has been deposited in the Protein Data Bank (2LE4). (**a**) Superposition of the 20 conformers that represent the solution structure of the 81-residue SOX2(39–118). The ordered region (residues 7–67 of the domain; 45–105 in the SOX2 numbering system) has a backbone RMSD of 0.74 Å. (**b**) Ribbon diagram of the ordered region (residues 7–67 of the domain; 45–105 in the SOX2 numbering system). Prior X-ray (5) and NMR (6) structures of the SOX2 DNA binding domain in complexes with other proteins have been published.
(TIFF)

Table S1 **Experimental details: protein sample, experimental conditions, NMR experiments, and orthogonal and tilted planes collected by ADAPT-NMR for a) SOX2, b) AeSCP2-PA, c) HSP12, d) RI-brazzein, and e) ubiquitin.**
(DOC)

Table S2 **Comparison of ADAPT-NMR with a pipelined approach consisting of 3D data collection, automated peak picking by SPARKY, and automated assignment by PINE-NMR.**
(DOC)

Table S3 **Sidechain assignment by ADAPT-NMR for proteins with available 3D sidechain spectra.** Peak lists from HCCH-TOCSY, HCCONH, HBHACONH, and CCONH spectra were provided to ADAPT-NMR.
(DOC)
