# Supplementary material for: Robust, Integrated Computational Control of NMR Experiments to Achieve Optimal Assignment by ADAPT-NMR
Source: PLoS One. 2012 Mar 12;7(3):e33173. doi: 10.1371/journal.pone.0033173 (PMC3299752; doi:10.1371/journal.pone.0033173)
Supplement: Text S1 — It discusses the robustness of the ADAPT-NMR approach and contains a complete description of the algorithm. (DOC) [file pone.0033173.s001.doc]

**On Robustness and Convergence of the Network**

Our network structure is a graph containing loops. Therefore, convergence to marginals is not guaranteed [1,2,3]. To overcome this, ADAPT-NMR’s multistep iterative approach utilizes dynamic graph topology, energy rescaling, and a variation of the basic belief propagation algorithm. The topology of the underlying graph *G = G(V,E)* that describes the factorization model is essential to achieving robust operation of the algorithm. In the initial stages, *G* does not include all combinations of pairs and triplets as part of the energy function, but only the combination of probabilistic variables with conditional dependencies above a given threshold. The algorithm starts with a subset of edges $A_{i}\subset E$that represent higher values of conditional dependencies. This reduces the loop complexity of the resulting graph and increases the probability of convergence. As additional data are collected and analyzed, the information in the system evolves, and some probabilistic variables and their marginal probabilities become effectively stationary so that edges with lower conditional dependencies can be added to graph *G*. The resulting changes in local topology, and the underlying factorization model, test the robustness of the “ground state” under local perturbations, which cause the reevaluation of less stable configurations while converged states remain stable.

**Complete Description of the Algorithm**

The iteration starts with the amino acid sequence of the protein and 2D NMR data from the two orthogonal planes (^1^H-^15^N, and ^1^H-^13^C). ADAPT-NMR employs tilted-plane data collection [4] and automated peak identification but combines this with probabilistic spin system generation. At first, on-the-fly evaluations of the spin systems determine which experiment and tilted plane should be collected next. The iteration continues until the spin system quality is good enough for initial calculations of sequence-specific assignments and secondary structure. Thereafter, an extended network, which takes into consideration spin systems, chemical shift assignments, and secondary structure, selects the next experiment and tilted plane. The iteration continues until the desired completeness of chemical shift assignments is achieved.

**Spectral Acquisition.** The optimum spectra (as determined in the optimization step) are collected by ADAPT-NMR, and are classified as *S_i,j_*, where ‘*i*’ is the experiment identifier (for example HNCA) and ‘*j*’ is the tilt angle. Tilted angle spectra are generally collected in pairs (*S_i,j_* and *S_i,-j_*).

**Spectral Processing.** The key derived measure in this step is the “conclusive probability” for each identified spectral peak, which is defined as the probability that a peak represents a real peak as opposed to an artifact or noise peak. ADAPT-NMR imports the most recently collected spectral data, co-registers all peaks by aligning all spectra, and peak picks spectra by an algorithm that assigns a probability to each peak on the basis of the noise level, peak intensity, the number of the residues in the protein, and the experiment type. The peaks are sorted by decreasing intensity. We represent the *k^t^*^h^ peak in the list as $P_{i,j}^{k}$, where ‘*i*’ stands for the experiment identifier and ‘*j*’ represents the tilted angle. Every 2D peak is an object adorned with various attributes (or properties), e.g., frequency coordinates, intensity, volume, possible back-projected 3D peak candidate, and priority weight. Newly identified 2D peaks are assigned initial values, which are updated in subsequent steps.

We generate a candidate 3D peak from every pair of peaks present in the 2D 0° and 90° planes of a given experiment that have a common ^1^H chemical shift (within a tolerance). The current 3D peak list is updated with tilted plane 2D peak data. Every 3D peak is an object with attributes (or properties): e.g., frequency coordinates 2D peaks projections in tilted and orthogonal planes, volume and intensity of its projection and their correlations, variation of projection coordinates, variation of projection priority weights, and possible associated spin systems. Probabilistic support vector machines [5] and modifications of Bayesian networks [6] form the basis of our machine learning algorithms. For each pair of planes *S_i,j_* and *S_i,-j_*, we determine whether each 3D peak candidate $C_{i}^{m}$ (peak in experiment ‘*i*’ with ‘*m*’ as the peak identifier) has a projection $P_{i,j}^{k}$ that corresponds to a peak in one of *S_i,j_* and *S_i,-j_*; if so, we flag $P_{i,j}^{k}$ as visited and update the attributes of $P_{i,j}^{k}$ and $C_{i}^{m}$. If any pair of peaks in *S_i,j_* and *S_i,-j_* having a common ^1^H chemical shift fails to correspond to a projection from the set of 3D peaks, we generate a new 3D candidate peak and initialize its list of attributes. We use several machine learning techniques simultaneously (a committee of machines) to derive statistical weights for the each peak’s attributes, and to derive its conclusive probability$CP_{i}^{m}$.

**Spin System Generation and Update.** An iterative, pseudo-energy, update algorithm derives probabilistic spin systems from available peak lists–this is one of the most complex sub-networks in ADAPT-NMR. The probabilistic network takes into account all attributes of the 3D peaks described above. Spin system objects are initialized from ^15^N-HSQC peaks and have multiple attributes and properties including eight fields that represent the chemical shifts of different classes of nuclei: *^13^C^α^_(i-1)_*, *^13^C^β^_(i-1)_*, *^13^C′_(i-1)_*, *^1^H_(i)_*, *^15^N_(i)_*, *^13^C^α^_(i)_*, *^13^C^β^_(i)_*, and *^13^C′_(i)_*, where *(i-1)* denotes the chemical shift of the previous residue. Each field is a probabilistic variable that might have multiple chemical shift choices. We represent spin system *‘k’* by *SS_k_,* and the field *‘i’* of spin system *‘k’* by $SS_{k}^{i}$(for example $SS_{6}^{4}$ means the ^1^H field of spin system number *6*). We denote the chemical shift choices of that field by $SS_{k}^{i}(j)$and their probabilities by $Pss_{k}^{i}(j)$where$\sum_{j} Pss_{k}^{i}(j) =1$*.*The chemical shift choices and their probabilities are calculated in the probabilistic network on the basis of 3D peak attributes, such as their resonance coordinates, volumes and/or peak heights, and conclusive probabilities. Other factors include the relative sensitivity of peak detection in different experiments (e.g., HN(CO)CA is more sensitive than HNCA for detecting *^13^C^α^_(i-1)_*, and in HNCA *^13^C^α^_(i)_* is more sensitive than *^13^C^α^_(i-1)_*), the variation of chemical shifts across experiments, and the agreement of ^13^C^α^ and ^13^C^β^ chemical shifts with those expected for the assigned amino acid type. A “null” state for matching is provided in order to represent the probability that no chemical shift in the data could be matched with the field. Null is a possible state for almost every probabilistic variable in ADAPT-NMR.

All attributes of spin systems are updated after each round of iteration and data collection. New spin systems are added if high probability peaks cannot be associated with any ^15^N-HSQC peaks. An important attribute of spin systems is “the probability of overlap”. In overlapped spectral regions, multiple spin systems may originate from a single ^15^N-HSQC peak. A probabilistic support vector machine (SVM) [5] has been trained to continuously evaluate the probability that the multiple chemical shift choices in a spin system object in fact represent more than one spin system. The number of choices, their probabilities, their corresponding 3D peak attributes, and eventually the assignment of the spin system are utilized to support the SVM decision. If the probability of overlap surpasses a threshold, the spin system is split. This feature of ADAPT-NMR has substantially improved the assignment quality of crowded spectral regions, and it is absolutely crucial for larger proteins.

**Update Assignment*.*** The quality of a spin system (*Q_i_)* is defined as:

$Q_{i}=\frac{\sum_{k} \max_{j} (Pss_{k}^{i}(j))}{Number of Spin Systems}$ (S1)

If the quality of a spin system is lower than a pre-selected threshold, the algorithm transfers control to the optimization step (described below). Otherwise, in the assignment step, probabilities for chemical shift assignments, secondary structure states, and outlier chemical shift values are determined. The core elements of this step were initially designed as part of the PINE algorithm [6]. However, extensions resulting from key insights gained from earlier work have resulted in modifications, including a fully probabilistic implementation of this step. The modifications, reflected in the ADAPT-NMR application, have led to substantial improvements in the quality of the assignment and secondary structure determination by providing notably improved estimates in two specific steps.

In the amino acid typing step, wherein given spin systems are scored against each amino acid residue in the protein, the enhancements in ADAPT-NMR protect against false positive assignments (for example, to noise peaks) in spin systems. Each resonance in a spin system is scored against all amino acids in the protein based on configuration probabilities (assignments and secondary structure), and the probability distribution of chemical shifts derived from BMRB. The probability that nucleus *‘i’* in spin system *‘k’,* matches residue *‘n’* in the protein is calculated as:

(S2)

where $p_{x}(n)$, the probability that chemical shift *‘x’* belongs to residue *‘n’* is calculated from:

$p_{X}(n)=\frac{p_{_{n}}(helix).g_{helix}^{n}(X)+p_{_{n}}(strand).g_{strand}^{n}(X)+p_{_{n}}(coil).g_{coil}^{n}(X)}{\sum_{j} p_{_{j}}(helix).g_{helix}^{j}(X)+p_{j}(strand).g_{_{strand}}^{j}(X)+p_{_{j}}(coil).g_{coil}^{j}(X)}$ (S3)

where$g_{helix}^{n}$*,* $g_{strand}^{n}$*,* and $g_{coil}^{n}$are the chemical shift probabilities of the related atom in residue *n* derived from BMRB and PDB databases, and $p_{n}(helix)$*,* $p_{n}(strand)$*,* and $p_{n}(coil)$are the secondary structure probabilities in the current iteration step*.* The cumulative score (probability) of a spin system *‘k’* against amino acid *‘n’* is calculated as:

(S4)

The second significant elements in the dynamic evolution of the assignment process are the connectivity score pseudo-energy terms in the factor graph model. The application in ADAPT-NMR of fully probabilistic constructs for deriving the connectivity terms is a major advance toward providing more accurate estimates than those achieved previously [6]. The connectivity score *ψ* of spin system *‘k’* and spin system *‘m’*, which measures the likelihood that they belong to consecutive amino acids in the protein sequence, is defined as:

(S5)

The equivalent pseudo-energy term can be calculated as:

$U_{i}(SS_{m},SS_{n})=-\frac{1}{\beta}\log\psi(SS_{m},SS_{n})$ (S6)

where ‘*f*’ measures the distance score between chemical shifts $SS_{m}^{i}(j)$and :

(S7)

The distance function denoted by *d* is the Euclidean distance, and *C* is determined by the spectral resolution. The default values for the constant *C* are 0.25 ppm for ^15^N and ^13^C, and 0.02 ppm for ^1^H. These values can be manually overridden.

**Optimization Step*.*** In the optimization step, ADAPT-NMR selects the next experiment and the next tilted plane for maximally informative data collection by utilizing information theory [7]. The first step is to use the level of “uncertainty” in the sense of information theory in order to identify the nuclei in the spin systems that are “weakest links” in the assignment process. This step pinpoints specific spin systems and candidate nuclei (fields) that have not been assigned uniquely and, therefore, have more “information uncertainty” (*IU*). In the next step we determine the optimal experiment that is expected to maximize the information gain. This step optimally selects the tilted plane with minimal overlap for the pinpointed peaks.

For each spin system nucleus *‘i’,* *IU* is calculated from:

$IU(i)=\sum_{k} H_{Assignment}(SS_{k})\cdot H_{Field}(SS_{k}^{i})$ (S8)

where *H_Assignment_* and *H_Field_* are Shannon entropy measures for the uncertainty in the assignment and the spin system field choices, respectively, as determined by

$H_{Assignment}(SS_{k})=-\sum_{i} P_{Assignment}^{SS_{k}}(i)\cdot\log\left(P_{Assignment}^{SS_{k}}(i)\right)$ (S9)

$H_{Field}(SS_{k}^{i})=-\sum_{j} Pss_{k}^{i}\left(j\right)\cdot\text{log}\left(Pss_{k}^{i}\left(j\right)\right)\text{ }$ (S10)

in which $P_{Assignment}^{SS_{k}}(j)$ reports the assignment probability that *SS_k_* belongs to residue *j* in the protein sequence. The probability is calculated in the *assignment step* of the process. In the first few rounds of iterations, where no assignments are available, *IU(i)* is calculated by setting the value of $H_{Assignment}(SS_{k})=1$ - i.e. a normalized maximal entropy state. The next data collection step seeks the experiment *‘E_i_’* with the highest “information content” *IC* as estimated from

$IC(E_{i})=\frac{\sum_{j\in F_{i}} IU(j)}{\left|F_{i}\right|}+k_{i}\cdot n_{i}$ (S11)

where *F_i_* is the index of nuclei that experiment *E_i_* covers*.* For example, HNCA covers *^13^C^α^_(i-1)_* and *^13^C^α^_(i)_*, which correspond, respectively, to nuclei 1 and 6. *|F_i_|* is the cardinality of the index set *F_i_,* *n_i_* is the count of tilted plane already collected for that experiment, and *k_i_* is derived empirically.

In order to select the optimum tilted plane in the selected experiment *E*, we seek the minimum for the following weighted overlap function:

where *‘i’* is the index for the dimension of the projection (in the case of projection into 2D planes $i\in\{1,2\}$), *d* is the standard Euclidean distance, and *C_i_* is proportional to the spectral resolution of each dimension. The following combined entropy measure $w_{C_{E}^{m}}$ is used to determine the uncertainty of peak$C_{E}^{m}$:

(S12)

where ‘*g*’ measures a “distance score” between the projection of 3D peaks *m* and *n* into plane *θ* as given by

$g(x,y)=\sum_{i} e^{-d(x_{i},y_{i})/C_{i}}$ (S13)

(S14)

where *A* is the set of spin systems associated with peak $C_{E}^{m}$ *,* $CP_{E}^{m}$ is the conclusive probability described above, and *F* is the index of nuclei covered by experiment *E.* Note that $w_{C_{E}^{m}}$is closely related to the *IU* and *IC* functions defined above. *IU* and *IC* find the weakest candidate nucleus by summing over all spin systems; however, $w_{C_{E}^{m}}$ achieves a more specific inference by summing over the spin systems that are potentially related to the peak$C_{E}^{m}$. Furthermore, $w_{C_{E}^{m}}$weights the sum in a manner that emphasizes peaks with higher uncertainty in the conclusive probability.

The selection set of tilted planes involves a search and prediction algorithm for all angles from 1º to 90º with increments of 1º, excluding planes that have already been collected. Data collection for the specified experiment and the specified tilted plane takes place without any user intervention.

**Evaluation Decision.** The latest results are generated (including the latest output files for chemical shift assignments, secondary structure, outliers, spin systems, 2D and 3D peak lists and their associated probabilities), and the overall assignment score is calculated (number of residues with assignment probability of one divided by the total number of residues). If this score falls below the specified target, the utility of further data collection is evaluated. Factors in this decision include whether the maximum numbers of tilted planes specified for each experiment have been reached, the quality factors, the number of residues, and the level of improvement in the assignment score from the previous iteration. If further data collection is not advised or if the assignment target score has been reached, a report is written, and the process terminates. Otherwise, data collection continues.

**REFERENCES**

1. Huang C, Darwiche A (1996) Inference in Belief Networks: A Procedural Guide. Int. J Approximate Reasoning 15:225-236.
2. Smyth P (1998) Belief networks, hidden Markov models, and Markov random fields: a unifying view. Pattern Recognition Letters 18:1261-1268.
3. Tatikonda SC, Jordan MI (2002) Loopy belief propagation and Gibbs measures. Uncertainty in Artificial Intelligence. San Francisco, CA, USA: Morgan Kaufmann. pp. 493-500.
4. Eghbalnia HR, Bahrami A, Tonelli M, Hallenga K, Markley JL. (2005) High-resolution iterative frequency identification for NMR as a general strategy for multidimensional data collection. J Am Chem Soc 127: 12528-36.
5. Platt JC (1999) Probabilistic outputs for support vector machines and comparisons to regularized likelihood methods. Advances in Large Margin Classifiers. pp. 61-74.
6. Bahrami A, Assadi AH, Markley JL, Eghbalnia HR. (2009) Probabilistic interaction network of evidence algorithm and its application to complete labeling of peak lists from protein NMR spectroscopy. PLoS Comput Biol 5:e1000307.
7. Shannon CE (1948) A mathematical theory of communication. Bell System Technical Journal 27:379-423, 623-656
