# Supplementary figures and images for: Robust, Integrated Computational Control of NMR Experiments to Achieve Optimal Assignment by ADAPT-NMR
Source: PLoS One. 2012 Mar 12;7(3):e33173. doi: 10.1371/journal.pone.0033173 (PMC3299752; doi:10.1371/journal.pone.0033173)

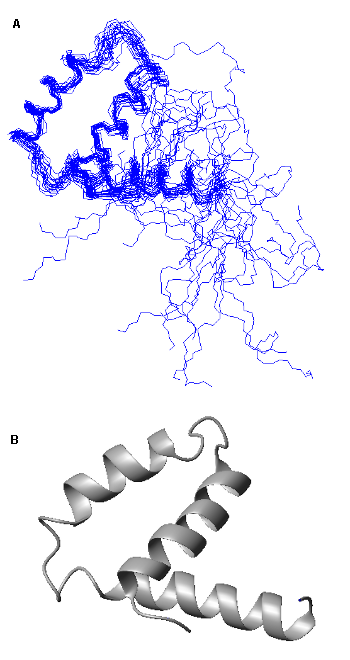

Supplement: Figure S1 — NMR solution structure of human SOX2(39–118). This structure has been deposited in the Protein Data Bank (2LE4). (a) Superposition of the 20 conformers that represent the solution structure of the 81-residue SOX2(39–118). The ordered region (residues 7–67 of the domain; 45–105 in the SOX2 numbering system) has a backbone RMSD of 0.74 Å. (b) Ribbon diagram of the ordered region (residues 7–67 of the domain; 45–105 in the SOX2 numbering system). Prior X-ray (5) and NMR (6) structures of the SOX2 DNA binding domain in complexes with other proteins have been published. (TIFF) [file pone.0033173.s002.tiff]
