# Supplementary material for: Robust, Integrated Computational Control of NMR Experiments to Achieve Optimal Assignment by ADAPT-NMR
Source: PLoS One. 2012 Mar 12;7(3):e33173. doi: 10.1371/journal.pone.0033173 (PMC3299752; doi:10.1371/journal.pone.0033173)
Supplement: Table S1 — Experimental details: protein sample, experimental conditions, NMR experiments, and orthogonal and tilted planes collected by ADAPT-NMR for a) SOX2, b) AeSCP2-PA, c) HSP12, d) RI-brazzein, and e) ubiquitin. (DOC) [file pone.0033173.s003.doc]

**Table S1. Experimental details: protein sample, experimental conditions, NMR experiments, and orthogonal and tilted planes collected by ADAPT-NMR for a) SOX2, b) AeSCP2-PA, c) HSP12, d) RI-brazzein, and e) ubiquitin**

1. SOX2(39–118) – HMG box (DNA binding domain) of the human stem cell transcription factor, which is essential to maintain self-renewal of undifferentiated embryonic stem cells.

Solution conditions: 0.71 mM protein, 10 mM MOPS buffer, pH 6.7. Data were collected at 15°C on a 600 MHz Varian VNMRS spectrometer with cryogenic probe.

| **SOX2** | 0° plane | | | Tilted planes | | | |
| --- | --- | --- | --- | --- | --- | --- | --- |
|  | # scans | #  increments | Time | Angles | # scans | #  increments | Time |
| HNCO | 4 | 64 | 11’4” | 15º, 27º, 75º | 4 | 64 | 21’29” |
| HN(CO)CA | 8 | 76 | 25’42” | 25º, 39º, 55º | 8 | 76 | 50’42” |
| HNCA | 8 | 76 | 25’10” | 14º, 22º, 29º, 41º, 48º, 58º | 8 | 76 | 49’41” |
| HN(CA)CO | 32 | 64 | 81’55” | 17º, 25º, 33º,41º,73º | 32 | 64 | 162’56” |
| CBCA(CO)NH | 16 | 63 | 41’23” | 17º, 27º, 36º, 45º, 53º, 66º | 16 | 63 | 82’8” |
| HN(CA)CB | 16 | 128 | 84’56” | 11º, 18º, 27º, 37º, 42º, 52º, 73º | 16 | 128 | 170’15” |
| ^15^N-HSQC  90° plane | 4 | 192 | 30’57” |  |  |  |  |

Timings:

50 h for tilted planes

5 h for 0° planes

55 h total

1. AeSCP-2-PA – sterol carrier protein from the mosquito *Aedes aegypti* complexed with palmitic acid (1:1 complex) (1).

Solution conditions: 2.0 mM protein, 10 mM phosphate buffer, pH 7.8. Data were collected at 25°C on a 600 MHz Varian VNMRS spectrometer with cryogenic probe.

| **AeSCP2-PA** | 0° plane | | | Tilted planes | | | |
| --- | --- | --- | --- | --- | --- | --- | --- |
|  | # scans | #  increments | Time | Angle | # scans | #  increments | Time |
| HNCO | 2 | 76 | 7’26” | 25º, 59º | 4 | 64 | 21’59” |
| HN(CO)CA | 8 | 96 | 32’53” | 35º, 38º, 59º | 4 | 96 | 32’50” |
| HNCA | 8 | 96 | 32’12” | 23º, 36º, 42º, 59º | 8 | 96 | 63’5” |
| HN(CA)CO | 16 | 64 | 43’22” | 27º, 42º, 53º, 74º | 16 | 64 | 85’22” |
| CBCA(CO)NH | 16 | 64 | 42’41” | 32º, 41º, 52º, 60º | 16 | 64 | 84’6” |
| HN(CA)CB | 16 | 128 | 84’34” | 24º, 35º, 46º, 51º, 54º, 64º | 16 | 128 | 168’45” |
| ^15^N-HSQC  90° plane | 2 | 192 | 16’15” |  |  |  |  |

Timings:

35 h for tilted planes

4 h for 0° planes

39 h total

1. HSP12 –Heat shock protein from *Saccharomyces cerevisiae* that helps cells survive stress conditions by protecting membranes against leaks and ruptures. This protein is naturally disordered in aqueous solution.

Solution conditions: 0.7 mM protein, 100 mM NaCl, 10 mM MOPS buffer, pH 7.0. Data were collected at 25°C on a 900 MHz Varian VNMRS spectrometer with cryogenic probe.

| **HSP12** | 0° plane | | | Tilted planes | | | |
| --- | --- | --- | --- | --- | --- | --- | --- |
|  | # scans | #  increments | Time | Angle | # scans | #  increments | Time |
| HNCO | 2 | 128 | 10’45” | 36º, 52º, 64º | 2 | 96 | 15’43” |
| HN(CO)CA | 2 | 96 | 8’14” | 22º, 38º, 53º | 2 | 96 | 15’49” |
| HNCA | 4 | 96 | 15’30” | 15º, 37º, 44º, 50º, 66º | 4 | 96 | 30’23” |
| HN(CA)CO | 4 | 96 | 16’37” | 20º, 39º, 51º, 61º | 4 | 96 | 31’20” |
| CBCA(CO)NH | 8 | 57 | 18’24” | 31º, 40º, 48º, 51º, 59º | 8 | 58 | 36’49” |
| HN(CA)CB | 4 | 128 | 20’58” | 16º, 41º, 56º, 63º, 70º | 4 | 192 | 61’44” |
| ^15^N-HSQC  90° plane | 4 | 192 | 29’58” |  |  |  |  |

Timings:

15 h for tilted planes

2 h for 0° planes

17 h total

1. RI-brazzein – A non-sweet mutant of the sweet protein brazzein from the plant *Pentadiplandra brazzeana* in which the dipeptide RI is inserted between L18 and A19 of the wild type protein (4).

Solution conditions: 1.0 mM protein, 10 mM phosphate buffer, pH 4.0. Data were collected at 37°C on a 600 MHz Varian VNMRS spectrometer with cryogenic probe.

| **RI-brazzein** | 0° plane | | | Tilted planes | | | |
| --- | --- | --- | --- | --- | --- | --- | --- |
|  | # scans | #  increments | Time | Angle | # scans | #  increments | Time |
| HNCO | 2 | 128 | 11’25” | 46º, 67º | 2 | 64 | 11’16” |
| HN(CO)CA | 4 | 96 | 17’8” | 38º, 48º | 2 | 64 | 11’48” |
| HNCA | 4 | 96 | 16’15” | 20º, 26º, 39º, 50º | 4 | 64 | 21’10” |
| HN(CA)CO | 8 | 96 | 32’18” | 37º, 49º, 66º, 76º | 8 | 96 | 63’9” |
| CBCA(CO)NH | 8 | 56 | 18’48” | 22º, 29º, 33º, 49º | 8 | 56 | 36’20” |
| HN(CA)CB | 8 | 128 | 42’7” | 9º, 25º, 38º, 61º, 67º | 8 | 96 | 62’26” |
| ^15^N-HSQC  90° plane | 4 | 256 | 41’50” |  |  |  |  |

Timings”

14 h for tilted planes

3 h for 0° planes

17 h total

1. Ubiquitin (*Chlorella*).

Solution conditions: 1.0 mM protein, 10 mM phosphate buffer, pH 6.6. Data collected at 25°C on a 600 MHz Varian VNMRS spectrometer with cryogenic probe.

| **Ubiquitin (*Chlorella*)** | 0° plane | | | Tilted planes | | | |
| --- | --- | --- | --- | --- | --- | --- | --- |
|  | # scans | #  increments | Time | Angle | # scans | #  increments | Time |
| HNCO | 4 | 96 | 16’54” | 25º, 55º | 2 | 64 | 11’38” |
| HN(CO)CA | 8 | 96 | 32’54” | 20º, 30º | 2 | 96 | 17’5” |
| HNCA | 4 | 96 | 16’45” | 16º, 36º, 81º | 4 | 96 | 32’13” |
| HN(CA)CO | 8 | 96 | 33’1” | 19º, 41º, 72º | 8 | 64 | 43’23” |
| CBCA(CO)NH | 8 | 64 | 21’59” | 15º, 35º, 45º | 4 | 64 | 21’59” |
| HN(CA)CB | 8 | 128 | 43’26” | 17º, 25º, 32º, 54º, 74º | 8 | 128 | 85’32” |
| ^15^N-HSQC  90° plane | 4 | 192 | 31’12” |  |  |  |  |

Timings:

13 h for tilted planes

2 h for 0° planes

15 h total

1. Ubiquitin (*Human*).

Solution conditions: 1.0 mM protein, 50 mM phosphate buffer, pH 6.5. Data collected at 25°C on a 600 MHz Varian VNMRS spectrometer with cryogenic probe.

| **Ubiquitin (human)** | 0° plane | | | Tilted planes | | | |
| --- | --- | --- | --- | --- | --- | --- | --- |
|  | # scans | #  increments | Time | Angle | # scans | #  increments | Time |
| HNCO | 4 | 96 | 17’ | 26º, 34º,  41 º | 2 | 64 | 12’ |
| HN(CO)CA | 8 | 96 | 33’ | 31º, 44º, 63º | 2 | 96 | 17’ |
| HNCA | 4 | 96 | 17’ | 22º, 44º, 55º, 67º | 4 | 96 | 32’ |
| CBCA(CO)NH | 8 | 64 | 22’ | 31º, 40º, 50º, 57º | 4 | 64 | 22’ |
| HN(CA)CB | 8 | 128 | 44’ | 11º, 42º, 61º, 67º | 8 | 128 | 86’ |
| ^15^N-HSQC  90° plane | 4 | 192 | 31’ |  |  |  |  |

Timings:

11 h for tilted planes

2 h for 0° planes

13 h total
