# Supplementary material for: Robust, Integrated Computational Control of NMR Experiments to Achieve Optimal Assignment by ADAPT-NMR
Source: PLoS One. 2012 Mar 12;7(3):e33173. doi: 10.1371/journal.pone.0033173 (PMC3299752; doi:10.1371/journal.pone.0033173)
Supplement: Table S2 — Comparison of ADAPT-NMR with a pipelined approach consisting of 3D data collection, automated peak picking by SPARKY, and automated assignment by PINE-NMR. (DOC) [file pone.0033173.s004.doc]

Table S2. Comparison of ADAPT-NMR with a pipelined approach consisting of 3D data collection, automated peak picking by SPARKY, and automated assignment by PINE-NMR

| Protein | Time for data collection and analysis by the pipeline method | Backbone assignment completeness/ accuracy by the pipeline method | Time for data collection and analysis by ADAPT-NMR | Backbone assignment completeness /accuracy by ADAPT-NMR |
| --- | --- | --- | --- | --- |
| HSP12 | 44 h | 93% / 96% | 17 h | 99% / 98% |
| AeSCP2-PA | 82 h | 96% / 97% | 39 h | 98% / 100% |
| RI-Brazzein | 33 h | 96% / 98% | 17 h | 98% / 100% |
| Ubiquitin (human) | 46 h | 95% / 97% | 15 h | 97% / 100% |
