# Supplementary material for: Robust, Integrated Computational Control of NMR Experiments to Achieve Optimal Assignment by ADAPT-NMR
Source: PLoS One. 2012 Mar 12;7(3):e33173. doi: 10.1371/journal.pone.0033173 (PMC3299752; doi:10.1371/journal.pone.0033173)
Supplement: Table S3 — Sidechain assignment by ADAPT-NMR for proteins with available 3D sidechain spectra. Peak lists from HCCH-TOCSY, HCCONH, HBHACONH, and CCONH spectra were provided to ADAPT-NMR. (DOC) [file pone.0033173.s005.doc]

Table S3. Sidechain assignment by ADAPT-NMR for proteins with available 3D sidechain spectra. Peak lists from HCCH-TOCSY, HCCONH, HBHACONH, and CCONH spectra were provided to ADAPT-NMR

| Protein | Data analysis time dedicated to sidechain assignment | Sidechain assignment completeness | Sidechain assignment accuracy |
| --- | --- | --- | --- |
| SOX2 | 15 min | 95% | 93% |
| RI-Brazzein | 7 min | 96% | 96% |
| Ubiquitin (human) | 10 min | 94% | 96% |
